# Making the case for azathioprine, methotrexate, and cyclosporine in active vitiligo management

**DOI:** 10.3389/fmed.2026.1744216

**Published:** 2026-06-02

**Authors:** Yan Valle, Julia Sigova, Maria Borodina, Lyailya N. Kayumova, Liliya I. Almakaeva, Vladimir B. Pinegin, Konstantin Lomonosov

**Affiliations:** 1 Vitiligo Research Foundation, New York, NY, United States; 2 Department of Dermatology, University of Studies Guglielmo Marconi, Rome, Italy; 3 Department of Neonatology, Faculty of Continued Medical Education, Pirogov Russian National Research Medical University, Moscow, Russia; 4 Russian Children's Clinical Hospital, Pirogov Russian National Research Medical University, Moscow, Russia; 5 Department of Dermatology and Communicable Diseases, Ministry of Health, I. M. Sechenov First Moscow State Medical University, Moscow, Russia

**Keywords:** azathioprine, corticosteroids, cyclosporine, JAK inhibitors, methotrexate, pediatric vitiligo, phototherapy, systemic immunosuppressants

## 1 Introduction

Despite growing evidence supporting the efficacy of azathioprine, methotrexate, and cyclosporine in halting vitiligo progression and promoting repigmentation, these agents remain underutilized in routine clinical practice. Current treatment paradigms overwhelmingly favor corticosteroids as the first-line systemic intervention, especially in cases of rapidly progressive or generalized disease. However, this corticosteroid-centric approach does not adequately address the chronic, relapsing nature of vitiligo and does not offer a sustainable long-term strategy for disease control.

Vitiligo affects approximately 0.76–1.11% of the US population, with up to 40% of cases potentially remaining undiagnosed (1). The condition imposes a substantial psychosocial burden comparable to other chronic inflammatory diseases, with patients experiencing depression, anxiety, stigmatization, and significant quality-of-life impairment (2).

Patients often cycle through topical agents and repeated corticosteroid mini-pulses, achieving only temporary stabilization at the cost of accumulating adverse effects. Meanwhile, immunosuppressants with decades of safety data in autoimmune conditions such as lupus, psoriasis, and inflammatory bowel disease remain relegated to off-label or last-resort use in vitiligo.

In our view, this discrepancy between available evidence and clinical practice represents a substantial treatment gap. We argue that azathioprine, methotrexate, and cyclosporine should no longer be relegated to off-label, last-resort use in vitiligo. Instead, they deserve structured, guideline-level consideration as systemic options for early disease stabilization and maintenance, particularly in patients for whom repeated corticosteroid pulses are unsafe, ineffective, or inaccessible. In this Opinion, we outline the rationale for this shift and propose a pragmatic path toward integrating these agents into routine care.

## 2 Reassessing systemic immunosuppression in vitiligo

The dominant model of “systemic therapy” in vitiligo has, in practice, meant repeated corticosteroid pulses. We believe this is too narrow. If the goal is durable disease control rather than crisis management, then other well-known immunosuppressants deserve to be reconsidered, not as exotic salvage options, but as planned elements of a long-term strategy. In this section, we revisit azathioprine, methotrexate, and cyclosporine through that lens.

### 2.1 Limitations of corticosteroid monotherapy

Corticosteroids, particularly in mini-pulse regimens, have demonstrated short-term efficacy in halting vitiligo activity. For instance, dexamethasone or betamethasone administered twice weekly over 3–6 months leads to disease arrest in approximately 80% of patients and repigmentation in 60% (3).

However, while such regimens are effective for short-term crisis management, they do not offer a sustainable strategy for a chronic, relapsing disease like vitiligo. The primary concern is not a single course, but the cumulative metabolic and systemic burden associated with repeated flare-and-retreatment cycles in routine care, including hypertension, hyperglycemia, and loss of bone density. Moreover, corticosteroids lack a defined role in long-term maintenance therapy, and many patients relapse following cessation. Furthermore, a significant proportion of patients, including those with comorbid diabetes, cardiovascular risk factors, or pediatric age, may be unsuitable candidates for sustained corticosteroid exposure. These limitations underscore the need for safer, maintenance-capable systemic therapies.

### 2.2 Azathioprine: immunomodulation with long-term utility

Azathioprine (AZA) is a purine analog that suppresses T-cell–mediated immunity and has long-standing use in dermatology and internal medicine. In vitiligo, randomized comparative trials have shown azathioprine (1–2.5 mg/kg/day) to be non-inferior to corticosteroid mini-pulse therapy in arresting disease activity, with equivalent efficacy by 6 months despite slower initial onset (4). A recent randomized controlled trial demonstrated that azathioprine combined with NB-UVB phototherapy achieved superior repigmentation and earlier stabilization compared to NB-UVB alone, with 88% of patients achieving disease arrest and significant reductions in vitiligo extent scores (5).

This slower onset is clinically important. In patients requiring immediate disease control, azathioprine is unlikely to function as an ideal rescue monotherapy. Its role may instead be better understood as that of a longer-term stabilizing agent, particularly in patients with recurrent activity or in those for whom repeated corticosteroid exposure is undesirable. In selected cases, a sequential approach may be considered, in which a faster-acting induction agent, such as a short course of systemic corticosteroids or cyclosporine, is used as a bridge while azathioprine reaches therapeutic effect.

Adverse events associated with azathioprine, principally gastrointestinal discomfort and reversible leukopenia, are usually infrequent and manageable at standard dermatologic doses. Pre-treatment screening for TPMT or NUDT15 deficiency, where available, can identify patients at higher risk of myelotoxicity and guide dose adjustment, but such pharmacogenomic testing is not yet standard of care worldwide. Regardless of testing status, routine laboratory monitoring (complete blood count and liver function tests at regular intervals) remains essential to detect hematologic or hepatic toxicity early.

While evidence in children with vitiligo is limited, azathioprine has been safely used in pediatric autoimmune disorders such as inflammatory bowel disease and juvenile dermatomyositis. Age-specific dosing based on body weight and vigilant laboratory monitoring are essential in these populations.

Nevertheless, vitiligo-specific studies remain limited by small sample sizes (*n* < 50), short follow-up durations (< 6 months), and heterogeneity in phototherapy protocols and outcome measures. Larger, multicenter trials are needed to establish optimal dosing, treatment duration, and long-term relapse rates.

Taken together, these data and decades of cross-indication experience lead us to view azathioprine as a rational candidate for long-term disease control in vitiligo, particularly in patients in whom chronic corticosteroid use is neither safe nor realistic.

### 2.3 Methotrexate: a familiar agent with emerging potential

Methotrexate (MTX), a folate antagonist used extensively in psoriasis and rheumatoid arthritis, has demonstrated disease-stabilizing effects in vitiligo at low weekly doses (7.5–15 mg) in small case series and observational reports. Clinical case series report significant repigmentation in patients with previously treatment-resistant vitiligo when treated with low-dose MTX (12.5–25 mg weekly), with several reports noting better responses as doses approach 20 mg weekly or higher (6). In patients with rheumatoid arthritis who developed vitiligo, MTX therapy resulted in both arthritis control and marked skin repigmentation.

Evidence for topical MTX formulations comes primarily from randomized controlled data. A randomized trial of topical 1% MTX gel demonstrated that MTX gel combined with NB-UVB phototherapy was more effective than either treatment alone, with the combination group showing the highest response rates and good to excellent therapeutic outcomes (7). However, topical MTX alone was not sufficiently effective to induce meaningful repigmentation as monotherapy.

In pediatric patients, MTX is widely used in juvenile idiopathic arthritis and pediatric psoriasis with well-established dosing protocols (typically 0.3–0.6 mg/kg/week). Though data in pediatric vitiligo are sparse, extrapolation from these indications supports cautious, weight-adjusted use with standard monitoring.

On this basis, we consider low-dose methotrexate a plausible and underused option for stabilizing active disease in carefully selected vitiligo patients, especially where systemic therapy is already contemplated for coexisting autoimmune disease.

### 2.4 Cyclosporine: a rapid-onset bridge therapy

Cyclosporine (CSA), a calcineurin inhibitor that blocks T-cell activation via NFAT inhibition, has been explored as an induction therapy in unstable vitiligo. In a randomized controlled study of 50 patients with active vitiligo, CSA 3 mg/kg/day achieved disease stabilization in 88% of patients compared to 84% with oral dexamethasone mini-pulse therapy (8). Critically, CSA demonstrated significantly faster onset to disease stabilization (10.9 weeks vs. 13.9 weeks, *p* = 0.01).

A recent Russian study of 40 patients with progressive vitiligo confirmed these findings, demonstrating that cyclosporine combined with NB-UVB 311nm phototherapy resulted in earlier stabilization of disease activity and more pronounced repigmentation compared to NB-UVB monotherapy (9). The combination therapy group also showed significant improvement in quality-of-life measures.

Due to its nephrotoxicity, hypertensive risk, and drug–drug interaction potential, CSA is generally reserved for short-term use, typically 3–6 months, with transition to safer maintenance agents once stabilization is achieved. Long-term cyclosporine data in vitiligo are limited, and treatment should be guided by careful monitoring of serum creatinine and estimated glomerular filtration rate, blood pressure control, and potential drug–drug interactions, with discontinuation or dose reduction if renal function declines or hypertension becomes difficult to manage.

Pediatric use of CSA is supported by experience in transplant medicine and atopic dermatitis, though vitiligo-specific data remain scarce. Age- and weight-adjusted dosing along with close monitoring are mandatory.

In our view, cyclosporine is best conceptualized not as a maintenance drug but as a short-term “bridge” therapy to rapidly arrest activity, allowing clinicians to transition patients to safer long-term regimens.

## 3 Discussion

We believe the evidence reviewed supports consideration of azathioprine, methotrexate, and cyclosporine as systemic options in selected patients with active vitiligo. This should not be interpreted as a call to replace corticosteroids broadly. According to current practice, short-course systemic corticosteroids remain the standard first-line systemic option for rapidly progressive or generalized active vitiligo because of their rapid onset and familiar short-term use profile (3, 4, 8). At the same time, the current treatment paradigm leaves limited room for longer-term stabilization strategies in patients with recurrent or persistent activity. In that context, conventional immunosuppressants may deserve more explicit consideration in carefully selected cases (4, 6, 8, 9).

### 3.1 Clinical positioning in active vitiligo

It is important to define the scope of this systemic approach. Azathioprine, methotrexate, and cyclosporine are not intended as substitutes for topical corticosteroids or topical calcineurin inhibitors in localized disease, where local therapy remains the preferred initial approach. Nor do we suggest that they replace systemic corticosteroids as the usual first-line option for rapidly progressive generalized vitiligo. Rather, their potential role is narrower: they may be considered in selected patients in whom systemic treatment is already warranted but the standard corticosteroid-centered approach is contraindicated, poorly tolerated, insufficiently durable, or impractical because repeated cycles are anticipated (3, 4, 6, 8).

This group may include patients with substantial metabolic risk, diabetes, hypertension, osteopenia, or pediatric considerations that make repeated corticosteroid exposure less desirable; patients who initially respond to corticosteroids but relapse promptly after withdrawal; and patients with recurrent generalized activity for whom a longer-term steroid-sparing strategy may be needed (3, 4, 6, 8, 9). In this sense, azathioprine, methotrexate, and cyclosporine are best viewed not as universal alternatives, but as selective systemic tools for a subset of active vitiligo patients.

### 3.2 A sequential strategy for selected patients

A common concern, particularly with azathioprine, is its slower onset of action relative to corticosteroids (4, 5). We agree that this limits its usefulness as sole rescue therapy when immediate disease arrest is required. For this reason, we do not propose azathioprine as a replacement for corticosteroids in acute rescue settings. However, in selected patients–especially those with early relapse after corticosteroid withdrawal or those likely to require repeated steroid cycles — a sequential strategy may be considered. In such cases, a rapid-acting induction agent, such as a short course of systemic corticosteroids or cyclosporine, may be used to control acute activity while a slower-acting agent such as azathioprine or methotrexate is initiated for longer-term stabilization (4, 8, 9).

This approach remains hypothetical in the absence of prospective vitiligo-specific maintenance trials and should be understood as a pragmatic clinical framework rather than an established treatment algorithm. Its rationale is to distinguish acute rescue from ongoing control in a chronic, relapsing disease, particularly when repeated corticosteroid exposure is becoming a recurring pattern rather than an isolated intervention (3, 4, 8).

### 3.3 Safety, access, and evidence limitations

One important consideration in the repositioning of these agents is their international accessibility. All three drugs are off-patent, manufactured by multiple generic producers, and listed on the WHO Model List of Essential Medicines for autoimmune indications (10). This availability contrasts with newer targeted therapies, which remain cost-prohibitive or inaccessible in many settings. Topical ruxolitinib, while an important therapeutic advance, is not a direct substitute for systemic stabilization in generalized active vitiligo and remains financially out of reach in many regions (11).

In terms of safety, these conventional immunosuppressants have well-documented and largely monitorable risk profiles. Azathioprine and methotrexate require laboratory monitoring for cytopenia and hepatotoxicity, and cyclosporine requires surveillance for nephrotoxicity and hypertension, but serious infections and malignancy are uncommon at standard dermatologic doses. By contrast, oral JAK inhibitors carry FDA and EMA boxed or class-wide safety warnings for serious infections, venous thromboembolism, lipid abnormalities, and malignancy risk, based on post-marketing safety signals (12). Topical ruxolitinib cream, which is currently the only FDA-approved drug for vitiligo, has not demonstrated systemic absorption-related toxicities in phase 3 trials and is labeled for limited body surface area involvement ( ≤ 10% BSA); its safety profile is therefore not directly comparable to that of systemic JAK inhibitors (11, 12). For selected patients, therefore, the appeal of azathioprine, methotrexate, and cyclosporine lies not in an absence of risk, but in the fact that their risks are familiar, monitorable, and may be acceptable when weighed against repeated corticosteroid exposure over time.

We also acknowledge the limitations of the current evidence base. Most available studies are small, often single-center, and of limited duration, with substantial heterogeneity in outcomes and treatment protocols (4–9, 13). Pediatric vitiligo-specific data remain sparse, and comparative effectiveness trials are rare (13). These limitations should encourage caution and better study design, not therapeutic inertia. In our view, the next step is not indiscriminate adoption, but clearer clinical positioning, prospective protocol development, and multicenter trials capable of clarifying which patients are most likely to benefit.

In conclusion, azathioprine, methotrexate, and cyclosporine should be viewed neither as universal replacements for corticosteroids nor as historical curiosities. Rather, they represent underused systemic tools with potential value in selected cases of active vitiligo, particularly when repeated corticosteroid exposure is unsuitable, poorly tolerated, or insufficient for durable disease control.
